# Changes in HIV‐1 Reservoir Dynamics After Mpox Infection

**DOI:** 10.1002/jmv.70690

**Published:** 2025-11-08

**Authors:** Guiomar Casado‐Fernández, Olivia de la Calle‐Jiménez, Inés Armenteros, Luis Lemus Aguilar, Daniel Fuertes, Juan Cantón, Elena Mateos, Noemi Cabello, Javier Rodríguez Añover, Anabel Negredo, Miguel Cervero, María Paz Sánchez Seco, Montserrat Torres, Vicente Estrada, Mayte Coiras

**Affiliations:** ^1^ Immunopathology and Viral Reservoir Unit, National Center of Microbiology Instituto de Salud Carlos III, Majadahonda Madrid Spain; ^2^ Infectious Diseases Unit, Internal Medicine Service Hospital Clínico San Carlos‐IdISSC Madrid Spain; ^3^ Healthcare Center Sandoval Hospital Clínico San Carlos‐IdISSC Madrid Spain; ^4^ Research in Medical‐Surgical Sciences Universidad Complutense de Madrid Madrid Spain; ^5^ Biomedical Sciences and Public Health Universidad Nacional de Educación a Distancia (UNED) Madrid Spain; ^6^ School of Telecommunications Engineering Universidad Politécnica de Madrid Madrid Spain; ^7^ Internal Medicine Service Hospital Universitario de Móstoles Madrid Spain; ^8^ Health Sciences, Faculty of Sciences Universidad de Alcalá, Alcalá de Henares Spain; ^9^ Biomedical Research Center Network in Infectious Diseases (CIBERINFEC) Instituto de Salud Carlos III, Majadahonda Madrid Spain; ^10^ Arbovirus and Imported Viral Diseases Unit, National Center of Microbiology Instituto de Salud Carlos III Madrid Spain; ^11^ School of Medicine Universidad Alfonso X El Sabio Madrid Spain; ^12^ Faculty of Medicine Universidad Complutense de Madrid Spain

**Keywords:** CD4+ coinfection, HIV‐1, lymphocyte activation, mpox, MPXV, T cells, viral reservoir

## Abstract

In May 2022, a large outbreak of monkeypox virus (MPXV) occurred in several non‐endemic regions worldwide. Another outbreak in 2024 raised global concerns, particularly for immunocompromised individuals like people with HIV (PWH). Since the latent HIV‐1 reservoir remains a major barrier to a cure, we investigated how past mpox infection affected reservoir dynamics in this population. PWH who had mpox at an average of 9 months before sampling showed a significantly smaller HIV‐1 reservoir than MPXV‐unexposed PWH. This reduction was accompanied by enhanced antigen‐driven proviral reactivation in CD4+ T cells, especially central memory cells (TCM), and increased expression of T‐cell activation and proliferation markers like CD32 and Ki67. Mpox also induced sustained immune stress, as reflected by a prolonged decrease in CD4+ T naïve (TN) cells, increased expression of immune senescence and exhaustion markers like PD‐1, LAG‐3, and CD57, as well as metabolic dysfunction in CD4+ TN and TCM cells, which showed impaired glucose uptake and reduced proliferative capacity. These findings suggest that MPXV‐induced immune activation leads to long‐lasting changes in T‐cell homeostasis and reprograms the HIV‐1 reservoir, which may have implications for monitoring immune competence and vaccine responses in this population. Understanding the interplay between HIV‐1/MPXV co‐infection, T‐cell dynamics, and HIV‐1 reservoir modulation provides novel insights into how controlled proviral reactivation may inform the design of cure‐oriented strategies.

## 1 Introduction

In May 2022, the monkeypox virus (MPXV) caused an outbreak in non‐endemic regions [1]. The countries with the most people infected were the United States, Brazil, and Spain [2], which accounted for 7332 confirmed infections, the highest absolute number in Europe, with most cases (95.7%) occurring in men who have sex with men (MSM) [3]. More recently, beginning in 2023 and escalating in 2024 with the emergence of a novel Clade Ib variant, a new mpox outbreak in Central Africa led to international spread, prompting WHO to declare a Public Health Emergency of International Concern in August 2024 [4]. Mpox infections continue to occur worldwide, maintaining ongoing public health concerns, particularly for immunocompromised populations. Mpox is a zoonotic disease that can be transmitted from animal to human and between humans. The main clinical manifestations include vesiculo‐pustular or skin rash and mucosal lesions, typically lasting 2–4 weeks [5]. While symptoms are generally self‐limiting, they can be painful, and immunocompromised individuals, such as people with HIV (PWH), experience higher rates of severe manifestations and complications, often requiring greater availability of specialized care and antiretroviral therapy programs [6]. Notably, during the 2022–2023 mpox outbreak, there was a strikingly high prevalence among PWH (38%–74%) [7, 8].

Viral coinfections can significantly impact HIV‐1 reservoir dynamics through multiple mechanisms, including immune activation, alterations in the levels of cellular subpopulations, and modulation of gene expression [9]. In particular, the activation and proliferation of latently HIV‐1‐infected T cells in response to distinct pathogens may disrupt the stability of the viral reservoir, contrasting with the homeostatic proliferation that typically maintains its constancy over time [10]. As a result of T‐cell activation, CD4+ T cells with increased capacity for glucose uptake and metabolism present a higher susceptibility to HIV‐1 infection and viral replication [11]. The inflammatory and metabolic responses triggered during immune activation may also induce the expression of key cellular factors involved in HIV‐1 transcription, such as NF‐κB. Moreover, HIV‐1 replication correlates with the phosphorylation of the innate antiviral factor SAMHD1 at Thr592 (pSAMHD1), leading to its inactivation and the consequent loss of its ability to impede viral propagation [12]. Therefore, antigen‐driven immune activation can potentially alter HIV‐1 latency and promote viral reactivation [13]. In parallel, the persistent presence of HIV‐1 contributes to chronic inflammation and immune system overload, which may accelerate immunosenescence in PWH [14], a process characterized by elevated levels of terminally differentiated cells expressing exhaustion and senescence markers like PD‐1 and CD57 [15].

The immune response to MPXV in humans remains poorly understood, with most insights derived from studies on other poxviruses [16]. MPXV infection elicits both innate and adaptive immune responses [17]. The innate response is marked by the rapid activation of Natural Killer (NK) cells and increased production of various cytokines. However, the cytokine profile, dominated by elevated levels of IL‐4, IL‐13, IL‐10, MIP‐1α/CCL3, and MIP‐1β/CCL4, suggests a CD4+ Th2 polarized response [18]. Simultaneously, low levels of IL‐2, TNF‐α, IFNα, and IFNγ indicate an anti‐inflammatory response likely mediated by regulatory T cells (Tregs) [19]. The humoral response is characterized by increased memory B cell proliferation and high levels of specific IgG. Notably, central memory and effector memory T cells, which are key contributors to the HIV‐1 reservoir, undergo rapid expansion during the early phase of mpox, while the naïve T‐cell subset decreases [20]. This decline could be critical for individuals with reduced CD4 counts who present an increased risk of severe complications and mortality due to infection, such as PWH with MPXV coinfection [20]. Mpox‐specific effector T‐cell responses may persist over a 2‐year timeframe postinfection [21].

Consequently, the profound immune activation produced during mpox could influence the size and composition of the HIV‐1 reservoir through several mechanisms such as direct viral reactivation driven by cellular activation, alterations in the CD4 subpopulations harboring the virus due to the renewed proliferative phase of most T‐cell subsets [17], and changes in the immunological environment that sustain viral latency.

In this study, we analyzed the impact of MPXV infection on the HIV‐1 reservoir landscape through a comprehensive analysis of peripheral blood T cell subpopulations, including their activation levels, glucose metabolic capacity, senescence and exhaustion markers, as well as their ability to support proviral reactivation. A deeper understanding of the effects of MPXV infection in PWH could enhance our knowledge of viral coinfection dynamics, contributing to improved management of HIV‐1 infection.

## 2 Methods

### 2.1 Study Participants

For this multicenter, observational, cross‐sectional study, we recruited 45 PWH between September 2021 and April 2023 from Hospital Clínico San Carlos (Madrid, España), Healthcare Center Sandoval (Madrid, Spain), Hospital Universitario Severo Ochoa (Madrid, Spain), Hospital Universitario La Paz (Madrid, Spain), and Hospital Clínic (Barcelona, Spain). Twenty‐four individuals with a documented MPXV infection (mpox+) were identified from medical records at participating centers and invited to join the study, while 21 participants without any history of exposure to MPXV, recruited in 2021 before the emergence of the outbreak in non‐endemic regions, served as controls (mpox−) selected to have similar demographic and clinical characteristics (Supporting Information S1: Table 1). Inclusion criteria required participants to be over 18 years old, have a CD4 count > 500 cells/μL at the time of enrollment, and be undergoing standard antiretroviral therapy (ART). For the mpox+ cohort, an additional criterion was the presence of signs or symptoms of mpox during physical examination at the time of infection, confirmed subsequently by a generic real‐time PCR for the Orthopoxvirus genus [22] and a generic conventional validated nested PCR for confirmation [23]. All mpox+ cases corresponded to mild disease, with general symptoms such as fever, myalgia, headache, asthenia, odynophagia or cough, and typical mpox manifestations including vesicles, rash, lymphadenopathy, proctitis or keratitis (Supporting Information S1: Table 1). None of the cases required hospitalization. None of the participants had received vaccination against mpox.

### 2.2 Ethical Statement

All participants provided written informed consent before inclusion in the study. The anonymity and confidentiality of all participants were safeguarded in compliance with current Spanish and European Data Protection Regulations. The study protocol (CEI PI 70_2022) adhered to the principles outlined in the Declaration of Helsinki and received prior approval from the Ethics Committee of the Instituto de Salud Carlos III (IRB IORG0006384), as well as the Ethics Committees of the participating centers.

### 2.3 Cells

Whole blood was collected in EDTA Vacutainers tubes (Becton Dickinson, Madrid, Spain) and processed immediately. Peripheral blood mononuclear cells (PBMCs) and plasma were isolated using Ficoll‐Hypaque density gradient centrifugation (Pharmacia Corporation, North Peapack, NJ) and subsequently cryopreserved until further analysis. PBMCs were cultured in RPMI medium (Biowhittaker, Walkersville, MD) supplemented with 10% (v/v) fetal bovine serum, 2mM l‐glutamine, 100 U/mL penicillin, and 100 µg/mL streptomycin (Gibco, Grand Island, NY). Due to limited sample availability, not all assays could be performed for every participant.

### 2.4 Determination of HIV‐1 Reservoir Size

Genomic DNA was extracted using the QIAamp DNA Blood Mini Kit (Qiagen Iberia, Madrid, Spain). Integrated HIV‐1 proviral DNA was quantified using a nested Alu‐LTR PCR assay [24]. Firstly, a conventional PCR was conducted on a StepOne Real Time Thermal Cycler (Thermo Fisher Scientific, Waltham, MA) with primers targeting Alu sequences and the HIV‐1 long terminal repeat (LTR). The thermal cycling conditions were: 95°C for 8 min; 12 cycles of 95°C for 1 min, 60°C for 1 min, and 72°C for 10 min; followed by a final extension at 72°C for 15 min. Subsequently, a second round of PCR was performed with a QX200 AutoDG Droplet Digital PCR System (BioRad, Hercules, CA) using TaqMan probes labeled with FAM/ZEN/Iowa Black (IDT, Integrated DNA Technologies, Coralville, IA) and the digital PCR supermix for probes (No dUTP) (BioRad). The *CCR5* gene served as the housekeeping gene to quantify input DNA and normalize the results.

### 2.5 HIV‐1 Proviral Reactivation

**Figure 1:**
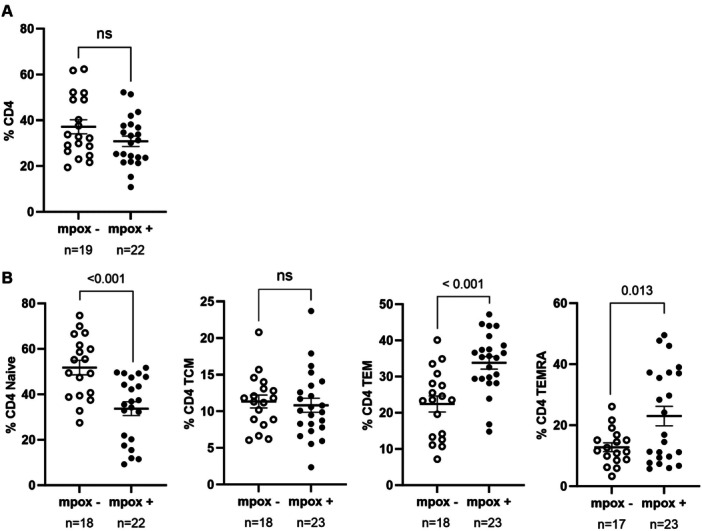
Total levels of CD4+T cells and distribution of CD4 memory subsets of PWH with (mpox+) and without mpox (mpox‐) recruited in Spain, September 2021‐April 2023. (A) Total CD4+ T cell levels in blood from participants from the PWH mpox+ and PWH mpox‐ cohorts. (B) Distribution of CD4+ T cell memory subsets in both cohorts. Each symbol represents one participant from each cohort: PWH mpox+ (closed circles) and PWH mpox‐ (open circles). Vertical lines correspond to mean ± standard error of the mean (SEM). Unpaired *t*‐test was applied to calculate the statistical significance between groups.

As previously described [25] CD4+ T cells were isolated using the CD4+ T Cell Isolation Kit (Miltenyi Biotec, Bergisch Gladbach, Germany) and then were incubated for 5 days with Dynabeads Human T activator CD3/CD28 (Thermo Fisher Scientific) and 300 U/ml IL‐2 (Chiron, Emeryville, CA). Phorbol 12‐myristate 13‐acetate (PMA) 25 ng/mL, ionomycin 1.5 μg/mL, and GolgiPlug Protein Transport Inhibitor containing Brefeldin A 1:1000 (BD Biosciences, San Jose, CA) were then added to the culture medium and incubated for 18 h. The cells were fixed and permeabilized using IntraPrep Permeabilization Reagent (Beckman Coulter, Spain) and subsequently stained with the following antibodies: CD45RA‐PE‐Cy7 and CD197/CCR7‐BV421 (BD Biosciences), p24‐Gag‐FITC (kc57 clone, Beckman Coulter, Spain), and pSAMHD1‐PE (SAMHD1 phosphorylated at T592) (Cell Signaling, Danvers, MA). The gating strategy is shown in Supporting Information S1: Figure 1. Data acquisition was performed in a BD LSRFortessa X‐20 flow cytometer using FACS Diva software (BD Biosciences), and data analysis was conducted with FlowJo software v10.0.7 (Tree Star Inc).

### 2.6 Glucose Uptake and Expression Levels of Glut‐1 Receptor

Glucose uptake and GLUT‐1 expression in CD4+ T cells were evaluated as markers of metabolic activity, given that metabolic reprogramming is closely linked to T‐cell activation, differentiation, and the ability of cells to sustain HIV‐1 infection [11]. PBMCs were incubated for 2 h in glucose‐free RMPI 1640 medium (Fisher Scientific), supplemented as previously described. To assess cellular glucose uptake capacity, the fluorescent glucose analog 2‐NBDG (2‐(N‐(7‐Nitrobenz‐2‐oxa‐1,3‐diazol‐4‐yl)Amino)‐2‐Deoxyglucose) (Thermo Fisher Scientific) was added to the culture medium at 30 µM, followed by a 10‐min incubation. Cells were then stained with the following conjugated antibodies: CD3‐BV510, CD4‐PE, CD45RA_PE‐Cy7, and CD197/CCR7‐BV421, to identify CD4 memory subsets; and GLUT‐1_AF647 (BD Biosciences). CD3+CD8‐ cells were classified as CD4+ T cells, including those with reduced CD4 expression due to HIV‐1 infection, as previously described [26]. Glucose uptake and GLUT‐1 receptor expression in CD4+ T cells were analyzed by flow cytometry, as explained above. The gating strategy is shown in Supplemental Figure 1.

### 2.7 Analysis of Immunosenescence and Exhaustion Markers

**Figure 2:**
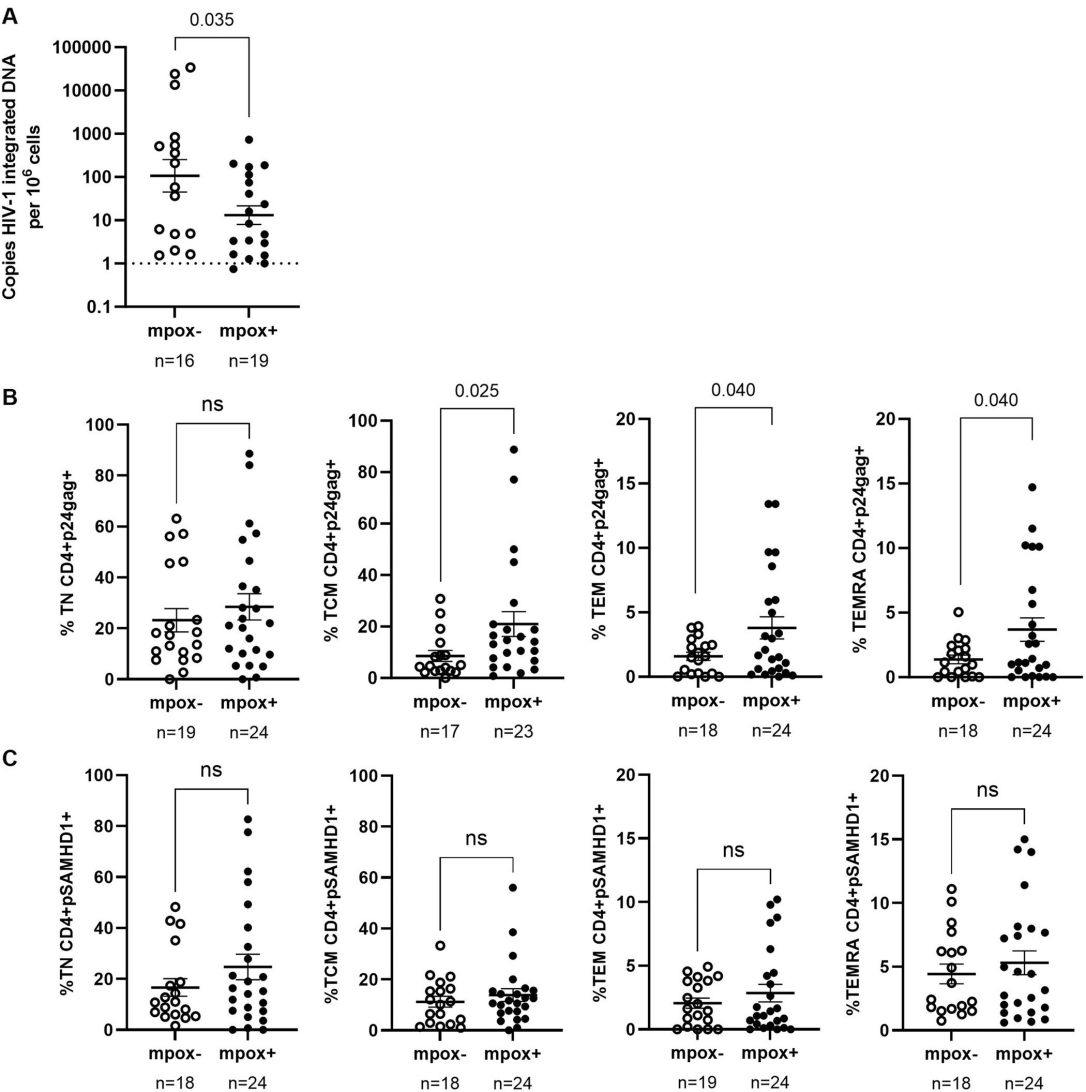
**A**nalysis of HIV‐1 reservoir size and proviral reactivation y CD4+ T cell memory subsets of PWH with (mpox + ) and without mpox (mpox‐) recruited in Spain, September 2021‐April 2023. (A) Copy number of HIV‐1 integrated DNA per million of PBMCs from PWH mpox+ and PWH mpox‐ cohorts, analyzed by ddPCR. (B) Proviral reactivation was analyzed by flow cytometry in isolated CD4+ T cells memory subsets from both cohorts after activation with anti‐CD3/CD28 microbeads for 5 days and subsequent activation for 18 h with PMA and ionomycin. (C) The levels of pSAMHD1 were also analyzed by flow cytometry in CD4+ T cell memory subsets after proviral reactivation. Each symbol represents one participant from each cohort: PWH mpox+ (closed circles) and PWH mpox‐ (open circles). Vertical lines correspond to mean ± SEM. Unpaired *t*‐test and Mann‐Whitney test were applied according to data normality to calculate the statistical significance between groups.

Immunosenescence and exhaustion markers were evaluated to determine whether mpox co‐infection may promote a senescent phenotype that could influence HIV‐1 persistence and immune competence. PBMCs were stained with Live/Dead Blue to discard dead cells and the following conjugated antibodies (Thermo Fisher Scientific): CD3‐NY660, CD8a‐NY730, CD45RA‐BUV661, and CD197/CCR7‐BUV737 to identify CD4 memory subsets; CD223/LAG‐3‐SB645, CD279/PD‐1‐SB780, and TIGIT‐AF700 to evaluate exhaustion markers; and KLRG‐1‐SB702, CD32‐PE‐Cy5.5, and CD57‐PE‐CYN7 to evaluate immunosenescence markers. Isotype controls were used to determine background signals. The gating strategy is shown in Supporting Information S1: Figure 2. Data acquisition was performed using a Cytek Aurora spectral flow cytometer, and data analysis was performed with FlowJo software v10.0.7 (Tree Star Inc., Ashland, OR).

### 2.8 Statistical Analysis

Statistical analyses were performed using GraphPad Prism software v10.1.2. (GraphPad Software Inc., San Diego, CA). Data normality was assessed with the Kolmogorov‐Smirnov test. For variables with parametric distribution, an unpaired t‐test was applied to compare group characteristics; for variables with non‐parametric distribution, the Mann–Whitney U test was used. Differences in sociodemographic characteristics between groups were evaluated using the chi‐square test. The correlation between p24‐gag and pSAMHD1 expression was analyzed across all CD4+ memory subsets using Spearman's rank correlation coefficient. The analysis was performed with Python, employing the Scikit‐Learn and Pandas libraries. The Seaborn library was used for the generation of regression plots. The Spearman correlation coefficient *r* was interpreted as follows [27]: values between ±0.40 and ±0.59, ±0.60, and ±0.79, or higher than ±0.8 were considered moderate, strong, or very strong correlations, respectively.

## 3 Results

### 3.1 Participants' Cohorts

Demographic and clinical characteristics of all participants are summarized in Table 1 and detailed in Supporting Information S1: Table 1. The median age was 39 years old (Interquartile range (IQR) 37–43.5) in the PWH mpox− cohort (*n* = 21) and 39.5 years old (IQR 35.3–46.5) in the PWH mpox+ cohort (*n* = 24) (*p* = 0.780). All participants were male. The median duration of HIV‐1 infection was 8 years (IQR 4.5–10.5) in the PWH mpox− cohort, with a median age at diagnosis of 32 years (IQR 27.5–37.5), while in the PWH mpox+ cohort, the median duration of infection was 9 years (IQR 6.3–10.8) (*p* = 0.270), with a median age at diagnosis of 30 years (IQR 26–35.8) (*p* = 0.392). The median CD4/CD8 ratio at the time of the study was 1 (IQR 0.7–1.4) in PWH mpox− and 1 (IQR 0.7–1.8) in PWH mpox+ (*p* = 0.845). All participants had an undetectable HIV‐1 viral load at the time of sampling, except for one individual in the PWH Mpox‐ group (4.8%) who exhibited detectable viremia due to ART interruption (*p* = 0.467). The most common ART regimen in both cohorts consisted of one integrase inhibitor (INI) plus one nucleoside reverse transcriptase inhibitor (NRTI) (38.1% in PWH mpox− and 45.8% in PWH mpox+ ; *p* = 0.764), followed by a regimen of one INI plus two NRTIs (42.8% and 25%, respectively; *p* = 0.226)).

**Table 1:** Demographic and clinical characteristics of PWH with (mpox+) and without mpox (mpox−) recruited in Spain, September 2021–April 2023.

	PWH mpox−	PWH mpox +	*p* value
	*n* = 21	*n *= 24	
Demographic characteristics			
Age, median (IQR)	39 (37–43)	39.5 (35.7–45.5)	0.780
Sex: male, *n* (%)	21 (100)	24 (100)	1
Clinical characteristics of HIV‐1 infection			
Time with HIV‐1 infection (years), median (IQR)	8 (5–10)	9 (6.7–10.2)	0.270
Median age at HIV‐1 diagnosis (IQR)	32 (28–37)	30 (26–35.2)	0.392
CD4/CD8 ratio, median (IQR)	1 (0.7–1.4)	0.7 (1–1.8)	0.845
CD4 count (cells/milliliter), median (IQR)	787 (653–971)	659.5 (575–867)	0.847
CD4 nadir (cells/milliliter), median (IQR)	410.5 (272.7–513.2)	336.5 (211.5–387.7)	0.325
CD8 count (cells/milliliter), median (IQR)	730 (607–900)	607 (487–918)	0.965
Viral load at the time of sampling, *n*, (%)			
Undetectable	20 (95.2)	24 (100)	0.467
Detectable (< 2.0 log)	1 (4.8)	0 (0)	0.467
Current ART regimen, *n* (%)			
2 NRTI + NNRTI	2 (10)	0 (0)	0.212
INI + 2 NRTI	9 (43)	6 (25)	0.226
INI + NNRTI	1 (5)	4 (17)	0.352
INI + NRTI	8 (38)	11 (46)	0.764
PI + 2 NRTI	1 (5)	1 (4)	> 0.999
2 NRTI	0 (0)	2 (8)	0.491
Clinical characteristics of mpox infection			
Time since mpox infection to sample (months), median (IQR)	—	8.6 (8.4‐9.3)	—

Abbreviations: HIV‐1, human immunodeficiency virus type 1; INI, integrase inhibitors; IQR, interquartile range; mpox, monkeypox; NRTI, nucleoside reverse transcriptase inhibitor; NNRTI, non‐nucleoside reverse transcriptase inhibitor; PI, protease inhibitors.

The PWH mpox+ cohort included individuals diagnosed with mpox between November 2022 and March 2023, none of whom had received prior mpox vaccination, and median time from mpox infection to sample collection was at an average of 8.6 months (IQR 7.8–9.3). The most common symptoms among participants were vesicles (9/24 participants; 37.5%), rash (4/24; 16.7%), fever (4/24; 16.7%), proctitis (4/24; 16.7%), and myalgia (3/24; 12.5%). Less frequent symptoms included lymphadenopathy (2/24; 8.3%), odynophagia (2/24; 8.3%), headache (2/24; 8.3%), cough (1/24; 4.2%), asthenia (1/24; 4.2%), pruritus (1/24; 4.2%), and keratitis (1/24; 4.2%).

### 3.2 Altered CD4+ T Cell Subset Distribution in PWH Mpox+ Cohort

No significant differences were observed in the total levels of CD4+ T cells between cohorts (Figure 1A). However, the PWH mpox+ cohort exhibited a 1.5‐fold reduction in CD4+ Naïve T (TN) cells (*p* < 0.001) compared to PWH mpox−, while CD4+ effector memory (TEM) and terminally differentiated (TEMRA) T cells were 1.5‐ (*p* < 0.001 = 0.0002) and 1.8‐fold (*p* = 0.013) higher, respectively, in PWH mpox+ compared to PWH mpox− (Figure 1B).

### 3.3 Reduced HIV‐1 Reservoir Size and Enhanced Proviral Reactivation in PWH Mpox+ Cohort

**Figure 3:**
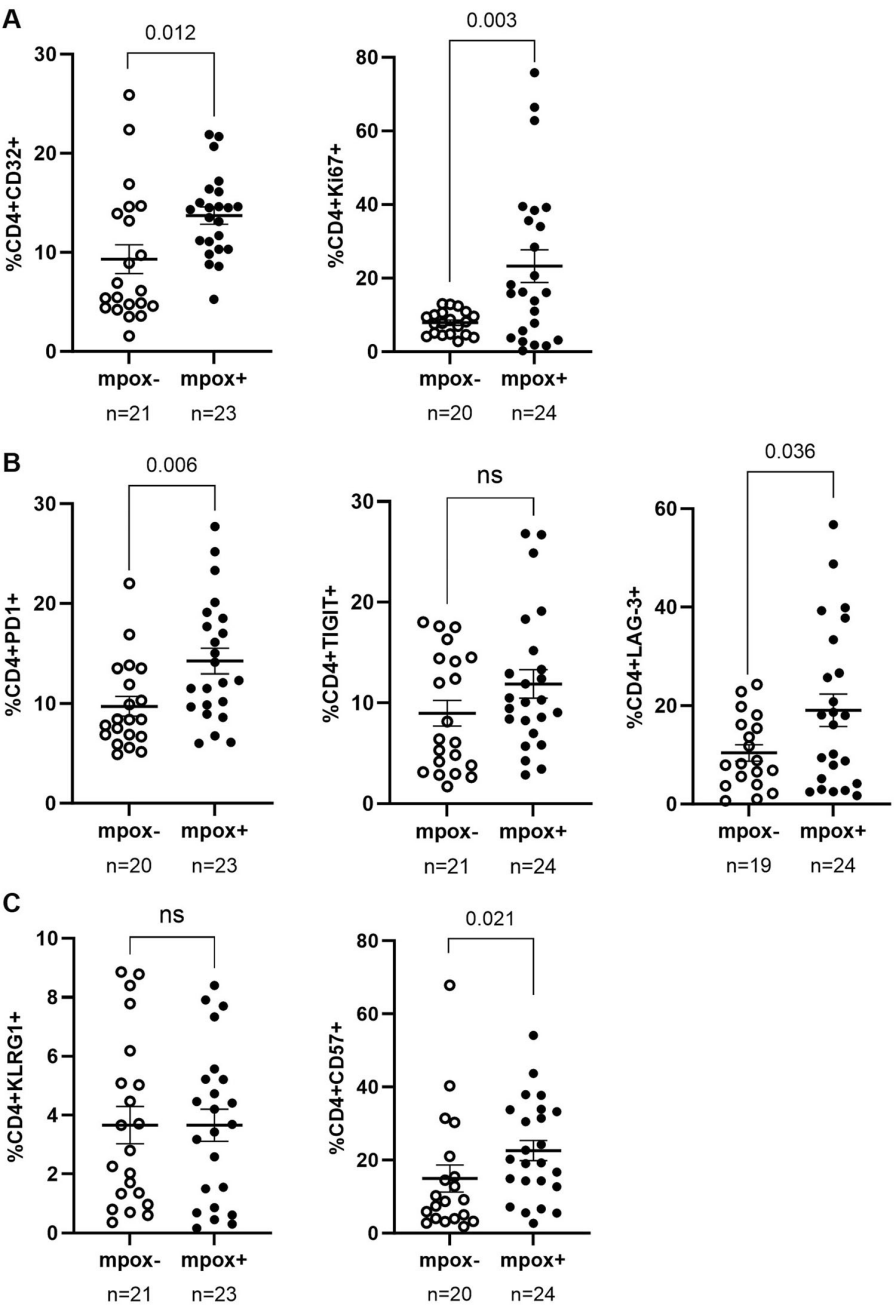
Analysis of the expression of activation and proliferation, exhaustion, and senescence markers in CD4+ T cells of PWH with (mpox + ) and without mpox (mpox‐) recruited in Spain, September 2021‐April 2023. The expression of activation and proliferation (A), exhaustion (B), and senescence (C) markers in CD4+ T cells from participants of PWH mpox+ and PWH mpox‐ cohorts was analyzed by flow cytometry. Each symbol represents one participant from each cohort: PWH mpox+ (closed circles) and PWH mpox‐ (open circles). Vertical lines correspond to mean ± SEM. Unpaired *t*‐test was applied to calculate the statistical significance between groups.

The HIV‐1 reservoir size, measured as integrated proviral DNA in the blood of individuals from the PWH mpox+ cohort, was 1.8‐fold lower compared to the PWH mpox− cohort (*p* = 0.035) (Figure 2A). Proviral reactivation in isolated CD4+ T cells was elevated in the PWH mpox+ cohort, with increases of 2.3‐fold (*p* = 0.025) in TCM, 2.3‐fold (*p* = 0.040) in TEM, and 2.7‐fold (*p* = 0.040) in TEMRA memory subsets (Figure 2B). Although no significant differences in the levels of pSAMHD1 were observed between the two groups in any CD4 memory subset (Figure 2C), the calculation of Spearman's rank correlation coefficient between the levels of p24‐gag and pSAMHD1 revealed significant positive correlations in all comparisons (Supporting Information S1: Figure 3). This correlation was stronger in both CD4+ T cell effector memory subsets TEM and TEMRA in the mpox+ cohort compared to the mpox− cohort (TEM: *r* = 0.56 vs. *r *= 0.73 (*p* < 0.01); TEMRA: *r* = 0.56 vs. *r* = 0.81 (*p* < 0.01), respectively), whereas it was similarly strong in CD4+ TN and TCM cells in both cohorts.

### 3.4 Increased Activation and Exhaustion Profiles in CD4+ T Cells of PWH Mpox+ Cohort

CD4+ T cells from the PWH mpox+ cohort exhibited significantly elevated activation marker levels, with CD32 being 1.5‐fold higher (*p* = 0.012) and Ki67 2.9‐fold higher (*p* = 0.003) compared to the PWH mpox− cohort (Figure 3A). Additionally, exhaustion markers PD‐1 (1.5‐fold; *p* = 0.006) and LAG‐3 (1.8‐fold; *p*=0.036) were also higher in the PWH mpox+ group (Figure 3B). The maturation marker CD57 also showed a 1.5‐fold increase (*p* = 0.021) in this cohort (Figure 3C).

### 3.5 Reduced Glucose Uptake and GLUT‐1 Expression in CD4+ T Cell Subsets of PWH Mpox+ Cohort

**Figure 4:**
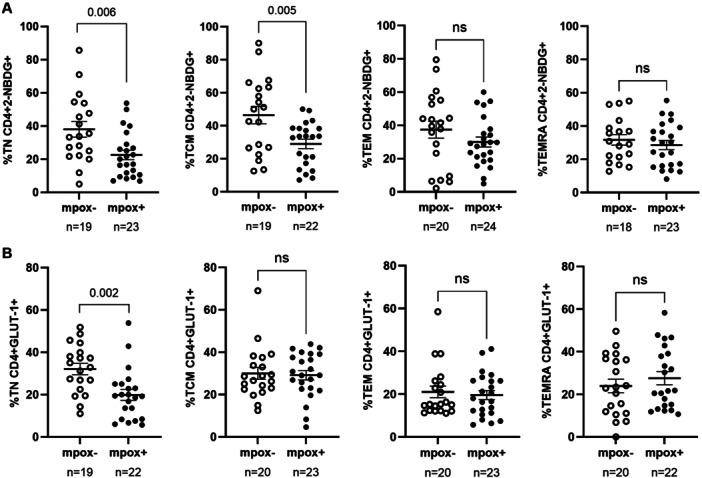
Capacity of CD4+ T cells to uptake glucose and expression levels of GLUT‐1 receptor of PWH with (mpox + ) and without mpox (mpox‐) recruited in Spain, September 2021‐April 2023. (A) The capacity of CD4+ T cells from participants of PWH mpox+ and PWH mpox‐ cohorts to uptake glucose was analyzed by flow cytometry using 2‐NBDG probe. (B) The expression levels of GLUT‐1 at the surface of these cells were also analyzed by flow cytometry. Each symbol represents one participant from each cohort: PWH mpox+ (closed circles) and PWH mpox‐ (open circles). Vertical lines correspond to mean ± SEM. Unpaired *t*‐test was applied according to data normality to calculate the statistical significance between groups.

Glucose uptake, measured with the 2‐NBDG probe, was 1.7‐ (*p* = 0.006) and 1.6‐fold (*p* = 0.005) reduced in CD4+ TN and TCM cell subsets from PWH mpox+ cohort, respectively, compared to PWH mpox− cohort (Figure 4A). Additionally, GLUT‐1 expression in CD4+ TN cells from PWH‐mpox+ cohort was reduced by 1.6‐fold (*p* = 0.002) compared to PWH‐mpox− cohort (Figure 4B).

## 4 Discussion

In this study, we demonstrate that prior mpox infection in PWH was associated with a significantly smaller HIV‐1 reservoir, accompanied by enhanced antigen‐driven proviral reactivation, particularly in central memory CD4+ T cells, when compared with PWH without prior mpox infection. These changes were observed in the context of sustained immune activation, as evidenced by increased expression of proliferation (CD32, Ki67), exhaustion (PD‐1, LAG‐3), and senescence (CD57) markers, together with a persistent loss of naïve and central memory CD4+ T cells, characterized by reduced glucose uptake and diminished proliferative capacity. Altogether, our data indicate that mpox co‐infection induces long‐lasting changes in CD4+ T‐cell homeostasis and impacts HIV‐1 reservoir dynamics, providing insights into how secondary viral infections may modulate reservoir persistence and immune competence in PWH.

PWH represent a distinct subpopulation due to the presence of CD4+ T cells that are latently infected with HIV‐1, a major barrier to achieving an HIV cure. This viral reservoir remains unchanged despite decades of effective ART and very slow decay has been documented in long‐term studies [28]. However, some qualitative changes may occur such as the selection for proviruses that are integrated in non‐inducible sites of the cellular genome [29] and cells that are resistant to cell death pathways [30]. Therefore, the high stability of the viral reservoir mostly relies on complex dynamics involving the homeostatic proliferation of infected CD4+ T cells driven by γc‐cytokines, insertional activation of particular host genes, and, mostly, normal responses to antigens through the T‐cell receptor (TCR) repertoire [28, 31]. However, the antigen‐driven proviral reactivation is usually followed by a contraction phase, and large clonal populations of HIV‐1‐infected CD4+ T cells may wax and wane in vivo [32].

Given these reservoir dynamics, we analyzed how mpox co‐infection in PWH may impact the long‐term stability of the HIV‐1 reservoir at an average of approximately 9 months after the acute infection with MPXV, since mpox−specific effector T‐cell responses may persist even 2 years after acute infection with MPXV [21]. It has been described that the levels of CD4+ T cells may decline during acute mpox infection, likely due to immune activation but also to immune tissue damage, such as the lymph nodes or thymus caused by MPXV replication [19]. This damage could explain the significant decrease in naive CD4+ T cells observed in our cohort of PWH, consistent with previous report [20], even at an average of 9 months after mpox infection. In addition, our participants exhibited higher levels of effector memory CD4+ TEM and TEMRA cells that are major contributors to the HIV‐1 reservoir [33]. The shift in CD4 count during acute mpox infection may result in a significant decrease in the CD4/CD8 ratio, a phenomenon previously observed even in HIV‐negative individuals [34]. However, we found no significant differences in the CD4/CD8 ratio in our mpox+ cohort compared to mpox− cohort, likely because the total levels of CD4 were similar between both cohorts despite the differences between subsets.

Beyond these phenotypic alterations, we observed profound functional changes in the HIV‐1 reservoir itself. By combining measurements of integrated proviral DNA with proviral reactivation, we provide a robust and complementary evaluation of the HIV‐1 reservoir in this cohort. Interestingly, the HIV‐1 reservoir size was significantly smaller in the cohort of PWH mpox+ compared to PWH nonexposed to MPXV. Moreover, the viral reservoir from PWH mpox+ showed a higher propensity for reactivation upon TCR‐mediated stimulation, particularly within CD4+ TCM cell subset, which serves as a major reservoir for HIV‐1 provirus through homeostatic regulation [35]. Similarly, the effector CD4+ TEM and TEMRA subsets, which are key contributors to reservoir replenishment, also showed increased susceptibility to reactivation. Supporting this enhanced reactivation phenotype, we also found a stronger correlation between the viral replication and the deactivation of the antiviral activity of SAMHD1 in the mpox+ cohort compared to mpox− cohort, supporting that the latently infected cells from the reservoir of Mpox+ cohort were in a higher activation state.

The observed reduction in reservoir size alongside enhanced reactivation capacity suggests that mpox infection may have initiated mechanisms leading to reservoir contraction. Several mechanisms may be involved in this reduction of the proviral reservoir size in the cohort of PWH mpox+ following TCR‐mediated activation of HIV‐1‐infected CD4+ T cells. First, the natural contraction of the immune response after the antigen wanes driven by homeostatic regulation may eliminate the HIV‐1‐infected CD4+ T cells reactivated from latency [19, 32]. Second, the induction of a latent HIV‐1 provirus, followed by the transcription and expression of viral proteins, may lead to cell death due to cytopathic effects, considering that most productively infected cells have a very short in vivo half‐life of approximately 1 day [32, 36]. And third, the proviral reactivation may also enable immune clearance of infected cells by cytotoxic cells such as HIV‐1‐specific CD8 + T cells and Natural Killer (NK) cells [37]. This concept underlies the “shock and kill” strategy, which aims to eliminate the HIV‐1 provirus by using latency‐reversing agents (LRAs) that induce viral reactivation and render infected cells visible to the immune system [38].

This overactivation of the immune system that occurred during MPXV acute infection and continued at least 8 months postinfection, was supported by the persistently increased expression of proliferation markers such as CD32 and Ki67 in CD4+ T cells from PWH mpox+ cohort. CD32, an Fc‐gamma receptor IIa (FcγR‐IIa), has been associated with activated CD4+ T cells [39] and its persistent expression has been reported in CD4+ and CD8 + T cells of individuals with chronic infections such as hepatitis B [40]. Reactivation of the latent HIV‐1 reservoir in ART‐treated PWH has been linked to an expansion of infected CD32+ cells that are resistant to NK cell‐mediated killing due to evasion of the antibody‐dependent cell cytotoxicity (ADCC) mechanism [41]. On the other hand, Ki67 is a nuclear protein expressed during cell proliferation [42] and its increased expression in CD4+ T cells has been associated with the clonal expansion and maintenance of latently infected cells, contributing to the persistence and stability of the HIV‐1 reservoir despite long‐term ART [28].

However, the observed reduction in reservoir size, driven by sustained CD4+ T cell activation, was not achieved without consequences. CD4+ T cells from our PWH mpox+ cohort exhibited elevated levels of exhaustion and senescence markers such as PD‐1, LAG‐3, and CD57. Consistently, HIV‐1 proviral DNA is preferentially harbored by CD4+ T cells expressing immune activation and proliferation markers such as Ki67 and PD‐1 [28]. T‐cell exhaustion is well‐documented in PWH and results from chronic immune activation driven by the persistent HIV reservoir, ultimately contributing to adverse health outcomes [43]. The upregulation of inhibitory receptors such as PD‐1 [44] and LAG‐3 [45] has been linked to disease progression, increased comorbidities, and poor outcomes [46]. Additionally, the overexpression of CD57 is associated with cellular senescence and marks highly differentiated, antigen‐specific T cells with shorter telomeres [47]. While elevated CD57 expression has been previously reported in CD4+ T cells from ART‐treated, well‐controlled PWH [48], the significant increase observed in our PWH mpox+ cohort suggested heightened immune system pressure, likely due to previous MPXV infection.

These immune perturbations extended beyond surface markers to affect fundamental cellular metabolism. Immune senescence in PWH is associated with reduced glucose uptake in CD4+ T cells due to mitochondrial dysfunction and GLUT1 downregulation, driven by chronic immune activation [49]. In our study, CD4+ TN and TCM cells from the PWH mpox+ cohort presented a reduced capacity to uptake glucose. Upon T‐cell stimulation, naive T cells undergo metabolic reprogramming, shifting towards glycolysis to generate the energy required for activation and proliferation in response to antigenic challenge [50]. In our cohort, PWH mpox+ showed lower levels of CD4+ TN cells with a reduced proliferative capacity, as indicated by their impaired glucose uptake and reduced expression of the GLUT1 receptor. This suggested a compromised ability to respond to new immune challenges, likely due to the profound immune dysregulation caused by previous mpox infection. Despite the increased proviral reactivation capacity observed in CD4+ TCM cells from PWH mpox+ , these cells also showed reduced glucose uptake. This metabolic impairment may be linked to higher senescence and a diminished ability to sustain homeostatic or antigen‐driven proliferation. Given the crucial role of TCM cells in maintaining the HIV‐1 reservoir, this dysfunction could also contribute to the lower reservoir size observed in PWH mpox+ cohort compared to participants never exposed to MPXV. In contrast, effector CD4+ TEM and TEMRA cells showed a glucose uptake capacity like that of PWH nonexposed to MPXV. This aligns with previous findings indicating that TEM and TEMRA subsets, as key contributors to reservoir replenishment, remain highly susceptible to HIV‐1 infection [11].

One potential limitation of this study is that the analysis of peripheral blood samples may not fully represent the HIV‐1 reservoir dynamics in other anatomical compartments. In addition, our findings are limited to individuals with self‐limited mpox infection and CD4 counts > 500 cells/μL, as no participants in our cohort required hospitalization or presented with severe complications. This homogeneous clinical presentation restricts the extrapolation of our immunological findings to individuals with moderate‐to‐severe mpox disease and preserved immune responses, who may exhibit different immune response patterns. Furthermore, the relatively limited sample size and the cross‐sectional design, with samples collected at a single time point after mpox infection, may constrain the detection of subtler associations and preclude evaluation of longitudinal trends. Finally, the absence of participants with prior mpox vaccination limits the generalizability of our observations to this increasingly relevant population. However, despite these potential sources of bias, the observed trends were consistent and biologically plausible, supporting the robustness of our conclusions.

In conclusion, our findings provide novel insights into the impact of mpox infection on the highly stable HIV‐1 reservoir and immune response in PWH. The observed reduction in the HIV‐1 reservoir size in PWH mpox+ cohort suggests that antigen‐driven reactivation, coupled with homeostatic regulation, cytopathic effects, and enhanced clearance of infected cells, contributed to HIV‐1 reservoir depletion in blood. However, the immune stress induced by mpox also led to persistent T‐cell exhaustion and metabolic dysfunction, particularly in CD4+ TCM cells, which play a central role in maintaining the latent reservoir. These data highlight the complex interplay between immune activation, metabolic reprogramming, and reservoir dynamics in the context of viral co‐infections. Understanding these mechanisms could help design future strategies towards an HIV cure by leveraging controlled antigen‐driven reactivation in combination with immunomodulatory approaches to enhance cytotoxic clearance of infected cells while minimizing immune exhaustion.

## Author Contributions

Mayte Coiras, Montserrat Torres, Vicente Estrada, María Paz Sánchez Seco, and Anabel Negredo conceptualized the study. Olivia de la Calle‐Jiménez, Guiomar Casado‐Fernández, Montserrat Torres, and Mayte Coiras wrote the manuscript. Inés Armenteros, Juan Cantón, Javier Rodríguez Añover, Miguel Cervero, and Vicente Estrada selected and recruited the participants and collected the blood samples. Luis Lemus Aguilar, Elena Mateos, Olivia de la Calle‐Jiménez, and Guiomar Casado‐Fernández processed and stored all blood samples. Olivia de la Calle‐Jiménez and Guiomar Casado‐Fernández performed the analyses. Olivia de la Calle‐Jiménez, Guiomar Casado‐Fernández, Montserrat Torres, and Mayte Coiras performed the statistical analyses. Inés Armenteros, Juan Cantón, Miguel Cervero, and Vicente Estrada collected clinical data. María Paz Sánchez Seco, Anabel Negredo, Vicente Estrada, and Mayte Coiras provided funds for the study. All co‐authors read and approved the final version of the manuscript.

## Conflicts of Interest

The authors declare no conflicts of interest.

## Supporting information


**Supplemental Figure 1:** Gating strategy to analyze CD4+ T cells memory subpopulations (A), proviral reactivation (B), SAMHD1 phosphorylation (C), glucose uptake (D), and expression of GLUT‐1 (E) in PBMCs.

**Supplemental Figure 2:** Gating strategy to analyze by flow cytometry activation, exhaustion, and senescence markers in CD4+ T cells from PBMCs of the participants.

**Supplemental Figure 3:** Spearman correlation between the levels of pSAMHD1 and viral replication (p24‐gag) in the cohorts of participants without (A) or with (B) mpox previous infection. Spearman correlation coefficient r and p‐values were calculated using a combination of Python libraries. Regression plots were generated using the Seaborn library

**Supplemental Table 1:** Demographic and clinical data of PWH recruited for this study who were not previously in contact with mpox (A) or had been infected with mpox (B), in Spain, September 2021‐April 2023.

## Data Availability

The data that support the findings of this study are available from the corresponding author upon reasonable request.
