# Supplementary material for: Changes in HIV‐1 Reservoir Dynamics After Mpox Infection
Source: J Med Virol. 2025 Nov 8;97(11):e70690. doi: 10.1002/jmv.70690 (PMC12595786; doi:10.1002/jmv.70690)
Supplement: Supplementary file 1 — Supplemental Figure 1: Gating strategy to analyze CD4+ T cells memory subpopulations (A), proviral reactivation (B), SAMHD1 phosphorylation (C), glucose uptake (D), and expression of GLUT‐1 (E) in PBMCs. [file JMV-97-e70690-s002.pptx]

## Slide 1
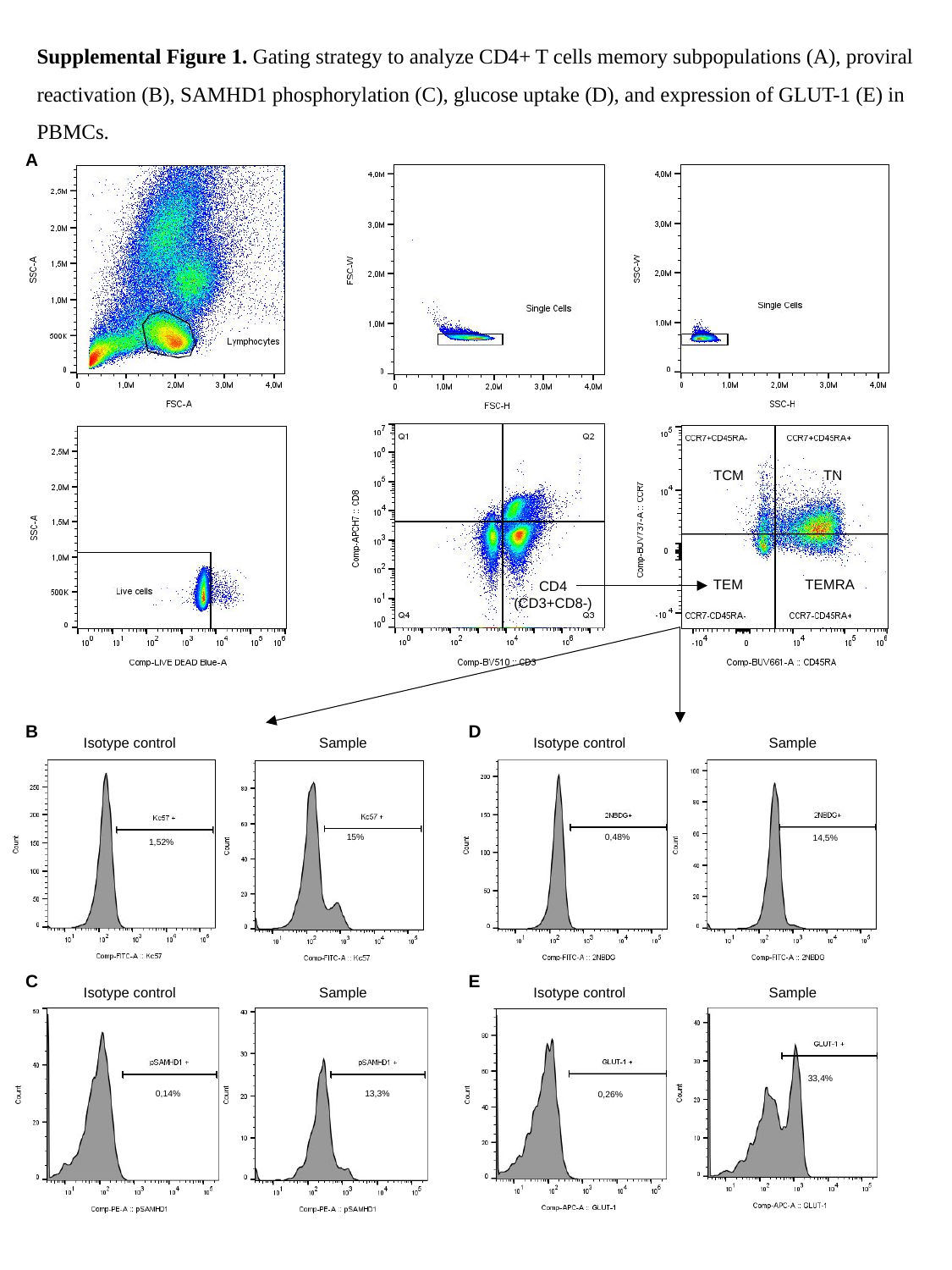

Supplemental Figure 1. Gating strategy to analyze CD4+ T cells memory subpopulations (A), proviral reactivation (B), SAMHD1 phosphorylation (C), glucose uptake (D), and expression of GLUT-1 (E) in PBMCs.
A
TCM
TN
TEM
TEMRA
CD4
(CD3+CD8-)
B
D
Isotype control
Sample
Isotype control
Sample
0,48%
15%
14,5%
1,52%
C
E
Isotype control
Sample
Isotype control
Sample
33,4%
13,3%
0,14%
0,26%
