# Supplementary material for: Changes in HIV‐1 Reservoir Dynamics After Mpox Infection
Source: J Med Virol. 2025 Nov 8;97(11):e70690. doi: 10.1002/jmv.70690 (PMC12595786; doi:10.1002/jmv.70690)
Supplement: Supplementary file 2 — Supplemental Figure 2: Gating strategy to analyze by flow cytometry activation, exhaustion, and senescence markers in CD4+ T cells from PBMCs of the participants. [file JMV-97-e70690-s001.pptx]

## Slide 1
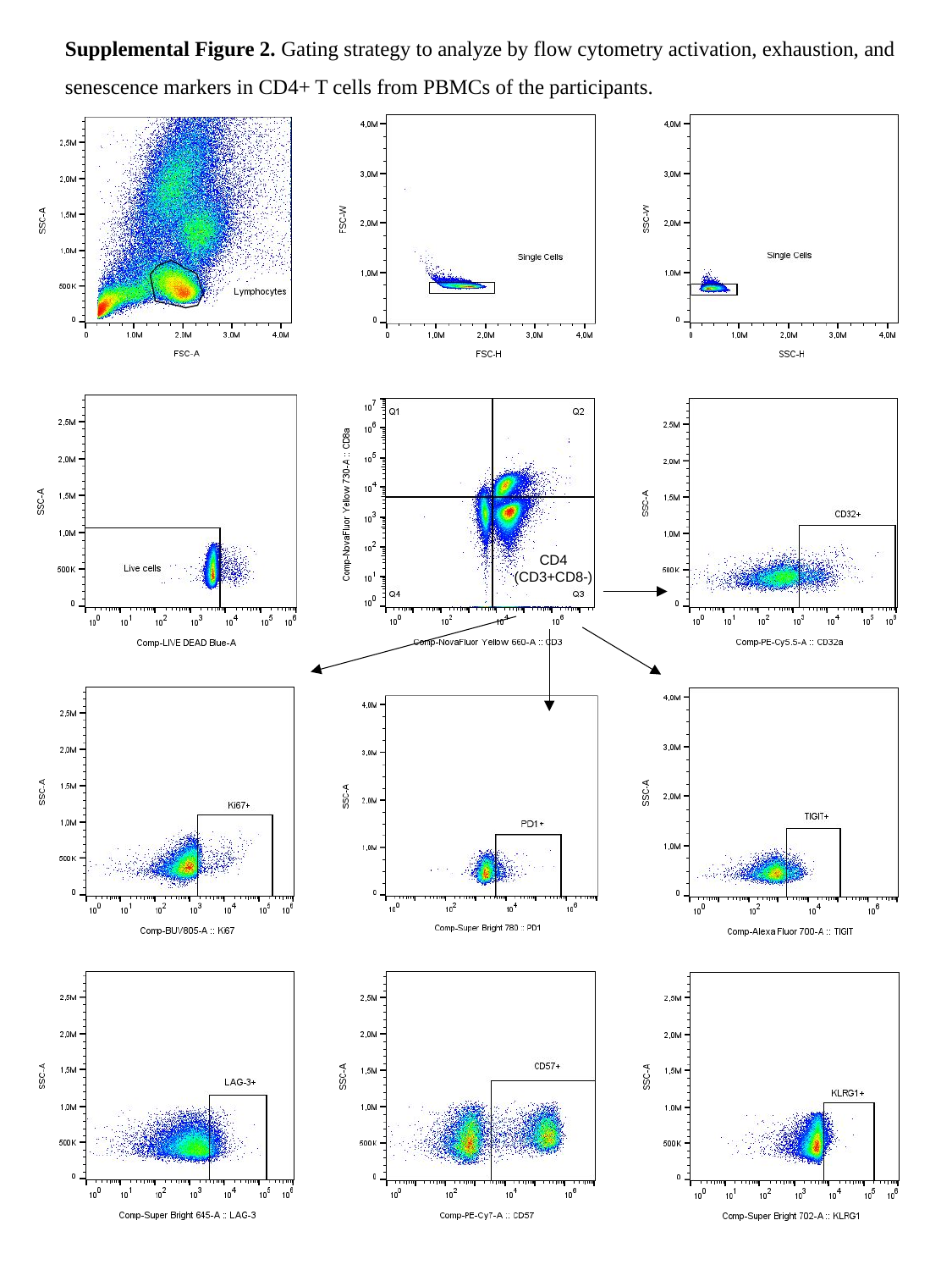

Supplemental Figure 2. Gating strategy to analyze by flow cytometry activation, exhaustion, and senescence markers in CD4+ T cells from PBMCs of the participants.
CD4
(CD3+CD8-)
