# Supplementary material for: Changes in HIV‐1 Reservoir Dynamics After Mpox Infection
Source: J Med Virol. 2025 Nov 8;97(11):e70690. doi: 10.1002/jmv.70690 (PMC12595786; doi:10.1002/jmv.70690)
Supplement: Supplementary file 3 — Supplemental Figure 3: Spearman correlation between the levels of pSAMHD1 and viral replication (p24‐gag) in the cohorts of participants without (A) or with (B) mpox previous infection. Spearman correlation coefficient r and p‐values were calculated using a combination of Python libraries. Regression plots were generated using the Seaborn library [file JMV-97-e70690-s004.pptx]

## Slide 1
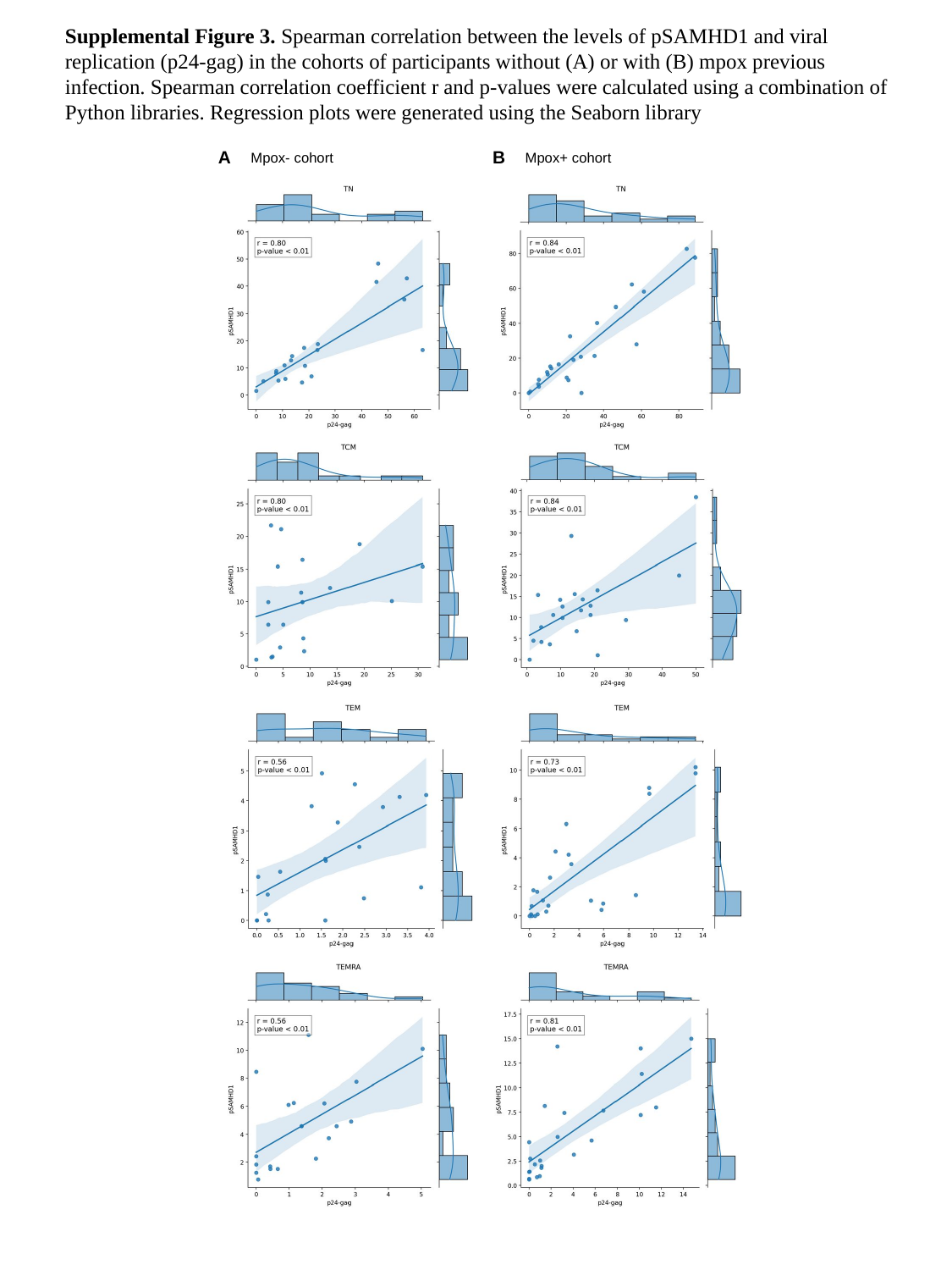

Supplemental Figure 3. Spearman correlation between the levels of pSAMHD1 and viral replication (p24-gag) in the cohorts of participants without (A) or with (B) mpox previous infection. Spearman correlation coefficient r and p-values were calculated using a combination of Python libraries. Regression plots were generated using the Seaborn library
A
B
Mpox- cohort
Mpox+ cohort
