# Supplementary material for: Changes in HIV‐1 Reservoir Dynamics After Mpox Infection
Source: J Med Virol. 2025 Nov 8;97(11):e70690. doi: 10.1002/jmv.70690 (PMC12595786; doi:10.1002/jmv.70690)
Supplement: Supplementary file 4 — Supplemental Table 1: Demographic and clinical data of PWH recruited for this study who were not previously in contact with mpox (A) or had been infected with mpox (B), in Spain, September 2021‐April 2023. [file JMV-97-e70690-s003.docx]

**Supplemental Table 1.** Demographic and clinical data of PWH recruited for this study who were not previously in contact with mpox (A) or had been infected with mpox (B), in Spain, September 2021-April 2023

**A)**

| **ID** | **Date of sampling** | **Age** | **Sex** | **Time since HIV diagnosis (years)** | **Age at HIV diagnosis** | **HIV Viral load at the time of sampling** | **Nadir CD4** | **CD4 count** | **CD8 count** | **Ratio CD4/CD8** | **ART** |
| --- | --- | --- | --- | --- | --- | --- | --- | --- | --- | --- | --- |
| 1 | 06/02/2023 | 46 | Male | 8 | 38 | Undetectable | 485 | 1101 | 607 | 1.81 | INI + NRTI |
| 2 | 07/02/2023 | 37 | Male | 4 | 34 | Detectable | 15 | 563 | 1111 | 0.51 | INI + NRTI |
| 3 | 07/02/2023 | 38 | Male | 9 | 30 | Undetectable | 426 | 787 | 730 | 1.08 | INI + 2 NRTI |
| 4 | 13/02/2023 | 48 | Male | 8 | 41 | Undetectable | 640 | 894 | 735 | 1.22 | INI + NRTI |
| 5 | 15/02/2023 | 39 | Male | 3 | 37 | unk | 305 | 535 | 673 | 0.8 | PI + 2 NRTI |
| 6 | 28/02/2023 | 43 | Male | 9 | 35 | Undetectable | NA | 298 | 490 | 0.61 | INI + 2 NRTI |
| 7 | 28/02/2023 | 43 | Male | 11 | 33 | unk | NA | 810 | 545 | 1.49 | INI + 2 NRTI |
| 8 | 29/03/2023 | 53 | Male | 8 | 45 | Undetectable | NA | 551 | 485 | 1.14 | INI + 2 NRTI |
| 9 | 30/01/2023 | 38 | Male | 13 | 26 | Undetectable | 414 | 977 | 659 | 1.48 | INI + NNRTI |
| 10 | 17/04/2023 | 52 | Male | 11 | 41 | Undetectable | NA | 784 | 783 | 1 | INI + NRTI |
| 11 | 21/03/2023 | 24 | Male | 1 | 24 | Undetectable | NA | 971 | 1632 | 0.59 | INI + NRTI |
| 12 | 15/09/2021 | 42 | Male | 4 | 38 | Undetectable | 613 | 897 | 449 | 2 | INI + 2 NRTI |
| 13 | 15/09/2021 | 38 | Male | 9 | 29 | Undetectable | 479 | 864 | 861 | 1 | INI + NRTI |
| 14 | 13/10/2021 | 35 | Male | 8 | 28 | Undetectable | 598 | 775 | 616 | 1.26 | INI + 2 NRTI |
| 15 | 15/09/2021 | 41 | Male | 10 | 31 | Undetectable | 245 | 1181 | 659 | 1.79 | INI + NRTI |
| 16 | 07/09/2021 | 40 | Male | 4 | 36 | Undetectable | 254 | 653 | 900 | 0.73 | INI + 2 NRTI |
| 17 | 16/11/2021 | 39 | Male | 12 | 27 | Undetectable | 320 | 665 | 1322 | 0.5 | INI + 2 NRTI |
| 18 | 15/09/2021 | 28 | Male | 10 | 19 | Undetectable | 868 | 998 | 1032 | 0.97 | NNRTI + 2 NRTI |
| 19 | 14/12/2021 | 24 | Male | 5 | 19 | Undetectable | 407 | 737 | 1600 | 0.46 | INI + NRTI |
| 20 | 08/09/2021 | 44 | Male | 15 | 30 | Undetectable | 279 | 1112 | 825 | 1.35 | NNRTI + 2 NRTI |
| 21 | 15/09/2021 | 37 | Male | 6 | 32 | Undetectable | 233 | 431 | 589 | 0.73 | INI + 2 NRTI |

ART: Antiretroviral Therapy; INI: Integrase Inhibitors; mpox, Monkeypox; NRTI: Nucleoside Reverse Transcriptase Inhibitor; NNRTI: Non-Nucleoside Reverse Transcriptase Inhibitor; PI: Protease Inhibitors.

**B)**

| **ID** | **Date of sampling** | **Age** | **Sex** | **Time since HIV diagnosis (years)** | **Age at HIV diagnosis** | **HIV Viral load at the time of sampling** | **Nadir CD4** | **CD4 count** | **CD8 count** | **Ratio CD4/CD8** | **ART** | **Time since mpox infection to sample (months)** | **Mpox main symptoms**† |
| --- | --- | --- | --- | --- | --- | --- | --- | --- | --- | --- | --- | --- | --- |
| 22 | 06/03/2023 | 47 | Male | 6 | 40 | Undetectable | 510 | 1386 | 759 | 1.83 | INI + NRTI | 9.27 | Vesicles, fever |
| 23 | 06/03/2023 | 31 | Male | 8 | 22 | Undetectable | 96 | 472 | 607 | 0.78 | INI + NRTI | 6.20 | Vesicles, fever, odynophagia |
| 24 | 08/03/2023 | 35 | Male | 8 | 26 | Undetectable | 340 | 476 | 487 | 0.98 | INI + NNRTI | 9.33 | Vesicles, myalgia, odynophagia |
| 25 | 08/03/2023 | 47 | Male | 15 | 31 | Undetectable | 363 | 629 | 914 | 0.69 | INI + NNRTI | 9.33 | Vesicles, cough, headache |
| 26 | 09/03/2023 | 34 | Male | 10 | 23 | Undetectable | 600 | unk | unk | unk | INI + NRTI | 8.37 | Vesicles, proctitis, fever |
| 27 | 10/03/2023 | 36 | Male | 5 | 30 | Undetectable | 351 | 837 | 476 | 1.76 | INI + NRTI | 8.40 | Vesicles, myalgia |
| 28 | 09/03/2023 | 47 | Male | 10 | 36 | Undetectable | unk | 552 | 583 | 0.95 | INI + 2 NRTI | 9.37 | Genital rash |
| 29 | 14/03/2023 | 37 | Male | 5 | 31 | Undetectable | 200 | 1300 | 700 | 2.4 | INI + 2 NRTI | 8.53 | Vesicles |
| 30 | 13/03/2023 | 39 | Male | 2 | 36 | Undetectable | unk | 1257 | 506 | 2.49 | INI + 2 NRTI | 9.50 | unk |
| 31 | 13/03/2023 | 40 | Male | 9 | 30 | Undetectable | unk | 598 | unk | unk | INI + NRTI | 9.50 | Vesicles, proctitis, asthenia |
| 32 | 14/03/2023 | 37 | Male | 13 | 23 | Undetectable | 780 | 1134 | 918 | 1.24 | INI + NNRTI | 8.53 | Vesicles, rash |
| 33 | 13/03/2023 | 36 | Male | 11 | 24 | Undetectable | unk | 570 | unk | unk | 2 NRTI | 9.50 | unk |
| 34 | 16/03/2023 | 34 | Male | 5 | 28 | Undetectable | unk | unk | unk | unk | INI + NRTI | 8.60 | Lymphadenopathy |
| 35 | 16/03/2023 | 51 | Male | 9 | 41 | Undetectable | 333 | 726 | 352 | 2.06 | INI + NRTI | 8.60 | Fever, rash, pruritus |
| 36 | 16/03/2023 | 47 | Male | 9 | 37 | Undetectable | unk | 630 | 1260 | 0.5 | INI + 2 NRTI | 8.60 | Vesicles, myalgia |
| 37 | 29/11/2022 | 45 | Male | 5 | 40 | Undetectable | unk | 590 | 334 | 1.77 | INI + 2 NRTI | unk | Keratitis |
| 38 | 15/12/2022 | 41 | Male | 7 | 35 | Undetectable | unk | 689 | unk | unk | INI + NRTI | 5.70 | Fever, lymphadenopathy, rash |
| 39 | 15/12/2022 | 40 | Male | 10 | 31 | Undetectable | unk | 855 | unk | unk | INI + NNRTI | 4.23 | Lymphadenopathy, rash |
| 40 | 10/01/2022 | 42 | Male | 17 | 26 | Undetectable | 216 | 709 | unk | unk | 2 NRTI | 4.60 | Headache, rash |
| 41 | 07/02/2023 | 47 | Male | 19 | 29 | Undetectable | 252 | 871 | 1154 | 0.75 | INI + NRTI | unk | Proctitis |
| 42 | 14/02/2023 | 33 | Male | 7 | 27 | Undetectable | unk | 405 | 998 | 0.41 | PI + 2 NRTI | unk | Proctitis |
| 43 | 30/01/2023 | 39 | Male | 9 | 30 | Undetectable | 396 | 1242 | 2798 | 0.44 | INI + 2 NRTI | unk | unk |
| 44 | 13/02/2023 | 31 | Male | 9 | 23 | unk | 96 | 472 | 607 | 0.78 | INI + NRTI | unk | unk |
| 45 | 27/02/2023 | 45 | Male | 15 | 31 | Undetectable | 210 | 622 | 440 | 1.41 | INI + NRTI | unk | unk |

*ART: Antiretroviral Therapy; INI: Integrase Inhibitors; mpox, Monkeypox; ; NRTI: Nucleoside Reverse Transcriptase Inhibitor; NNRTI: Non-Nucleoside Reverse Transcriptase Inhibitor; PI: Protease Inhibitors; unk: unknown.

†Rash denotes any cutaneous eruption; vesicles denote a fluid-filled, elevated primary lesion ≤0.5–1 cm.
